# Irrigated barley–grass pea crop mixtures can revive soil microbial activities and alleviate salinity in desertic conditions of southern Morocco

**DOI:** 10.1038/s41598-023-40337-9

**Published:** 2023-08-14

**Authors:** Dennis S. Ashilenje, Erick Amombo, Abdelaziz Hirich, Krishna P. Devkota, Lamfeddal Kouisni, Abdelaziz Nilahyane

**Affiliations:** 1 African Sustainable Agriculture Research Institute (ASARI), Mohammed VI Polytechnic University (UM6P), Laayoune, Morocco; 2 Soil, Water, Agronomy (SWA) Program, International Center for Agricultural Research in the Dry Areas (ICARDA), Rabat, Morocco

**Keywords:** Biochemistry, Microbiology, Plant sciences

## Abstract

Soil salinity adversely limits crop and soil health, and this can be reversed by cropping systems where species exclude salts and activate microbial nutrient cycling. A randomized complete block design experiment was established in Laayoune–Morocco to evaluate the influence of irrigated grass pea and barley monocrops or combined together in 50–50% and 70–30% mixtures against soil salinity and CO_2_-C flux in sites with varying salinity. Site by treatment interaction significantly influenced (p < 0.05) soil salinity and CO_2_-C flux. Salinity reduced by 37 to 68 dS m^−1^ in highly saline soils across season regardless of treatment and barley monocrop retained the least salinity (15 dS m^−1^). Same applied to sites with low (1 to 2 dS m^−1^) and medium (2 to 5 dS m^−1^) salinity although less pronounced. The 70–30% grass pea, barley mixture maintained the greatest CO_2_-C flux in soils with low salinity and marginally enhancing soil active carbon (130 to 229 mg kg^−1^ soil) in different sites. Increasingly saline water filled pore space devastated CO_2_-C flux, although this process recovered under barley at extreme salinity. Overall, barley in mixture with grass pea can alleviate salinity and accelerate microbial carbon sequestration if irrigation is modulated in shallow desertic soils.

## Introduction

Arid lands cover 35% of the earth’s surface and support an estimated 20% of human population^1^. Requisite use of these lands for agricultural production demand that soil degradation be reversed and crop productivity enhanced. Perennially scarce precipitation (< 250 mm annum^−1^) hardly enough to support evapotranspiration and soil salinity are primary factors limiting plant growth in arid ecosystems^2^. Salinity and sodicity affect an estimated 340 million and 540 million ha of the total cultivated land worldwide respectively^3^. The adverse effects of aridity and salinity produce scanty or no vegetation in severe cases. This down plays assimilation of carbon and its availability to soil microbes. Therefore, reversing salinization can revitalize soil health.

Salinization in arid lands is characterized by accumulation of soluble elements including HCO_3_^−^, SO_4_^2−^, Cl^−^ and Na^+^ ions as a result of saline irrigation water or inadequate precipitation^4^. Hyperaccumulation of these elements can be toxic to plants. Salinity can co-occur with alkalinity, where accumulation and hydrolysis of Na and NaCO_3_ releases OH^−^ ions^5^. This effect elevates pH above 8.5 and consequently phosphorus, calcium, magnesium, zinc and ion precipitate to forms unavailable to plants. It gets worse when soils are both saline and sodic. Sodicity occurs when partial leaching of solutes from the top soil leaves Na^+^ versus Ca^+^ and Mg^+^ ion adsorption ratio in soil solution exceeding 6^5^. The aberrative effect of sodicity is a well elaborated topic^6^. Sodicity typically creates a low osmotic potential and therefore suppresses water uptake to levels that can disrupt plant and microbial functioning and development. The bacterial homeostasis in response to osmotic stress demands energy at the expense of cell growth and microbial activities. Soil microbial respiration can be a useful indicator of soil restoration from salinity.

Persistent salinity commonly associated with saline irrigation water can adversely affect soil physical properties and productivity. Soil particle dispersion on wetting and compaction after drying are prevalent in sodic and alkaline soils (pH > 9)^7,8^. Soil disaggregation exposes the organic carbon protected by clay minerals to mineralization^9^. The compacted soil layer obstructs drainage and as a result, the retained salts stunt crops^10^. Hence, it was of essence to interrogate ways to rehabilitate these soils and enhance crop ecosystem services of carbon sequestration and nitrogen cycling. Inevitably, and inhibitory effects of NaCl against soil microbial enzymes^11^, and limited turnover of crop residues and root activities restrain C and N cycling in saline soils^12^. The mirror of what happens in non-saline conditions is that organic acids secreted by crop roots provide active carbon as a source of energy for soil rejuvenating bacteria and fungi^13^. Soil microbes have profound mechanism that can amend the soil structure. For instance, polysaccharides secreted by bacterial biomass adheres to soil minerals and contribute to soil micro aggregation^14^. Fungi on other hand specialize in soil aggregation by their hyphae network^13^. These mechanisms have formed the basis for soil regeneration in non-saline soils. Nevertheless, we envisaged that, these benefits might be replicated in arid-saline conditions in association with annual cover crop species acclimatized to salinity. There is some light to this effect as^15^ recently demonstrated in saline-sodic soils that bacteria dominate nutrient cycling while fungi major in modifying the soil structure.

Soil microbial activity in cultivated systems is influenced by soil wetting and drying cycles, soil temperature and the abundance and quality of organic substrates in the soil. Alternate soil wetting and redrying can encourage CO_2-_C flush which has been explained by different theories related to availing of carbon substrates for mineralization. Microbial cells tend to lyse and release organic bound solutes on rehydration following a period of exposure to dry conditions^16^. Enzyme from the dead cells degrade the organic biomass and in the process enrich the media with labile carbon^17^. This together with disruption of mineral complex on rewetting exposes more carbon to mineralization. In reiteration of the adverse effects of salt toxicity, there is need to identify cropping systems that can balance mechanisms of microbial carbon mineralization with stabilization in soils. Such moderation has been observed in annual legume-grain crop mixtures in temperate conditions^18^. In this study, the goal was to combine a salt tolerant grain crop expected to extract sodium from the root zone of a community that includes an annual legume. It was envisaged that this synergy will enhance crop establishment and further support microbial activities to revitalize the structure and nutrient cycling of soils degraded by salinity. Hence, the aim of this study was to determine the influence of irrigated barley (*Hordeum vulgare*) and grass pea (*Lathyrus sativus*) monocrops and mixed crops on soil microbial respiration and investigate related chemical and physical mechanisms of rejuvenating soils from saline and desertic conditions. We specifically tested the hypothesis that barley, grass pea mixtures can alleviate salinity and enhance microbial activities of soil rejuvenation compared to their respective monocrops.

## Results

### Weather conditions prevailing during the period of gas measurements

**Figure 1:**
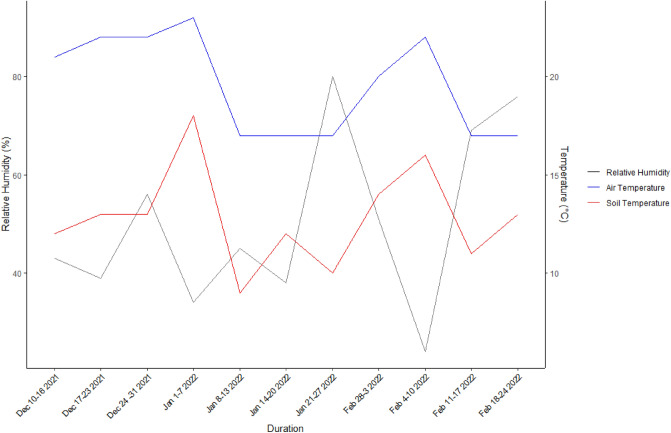
Relative humidity, air and soil temperatures averaged weekly during December 2021 to February 2022 in Foum El Oued, Laayoune, Morocco.

The relative humidity remained moderate from 43% in December 2021 and remained within a range of 34 and 54% upto the third week of January 2022 (Fig. 1). Subsequently the relative humidity increased sharply to 80% during the fourth week of January and fluctuated to a minimum of 24% in second week and rose back to76% at the end of February. Air temperature (averaged weekly) increased steadily during December and first week of January (21 to 23 °C) and remained at 17 °C in the rest of January. Air temperature increased again in the first two weeks of February to reach 22 °C but thereafter reduced to 17 °C. Soil temperatures were on average 7 °C lower, but followed a general pattern comparable to the air temperatures.

### Influence of barley–grass pea mixtures on soil CO_2_-C flux and active carbon

**Figure 2:**
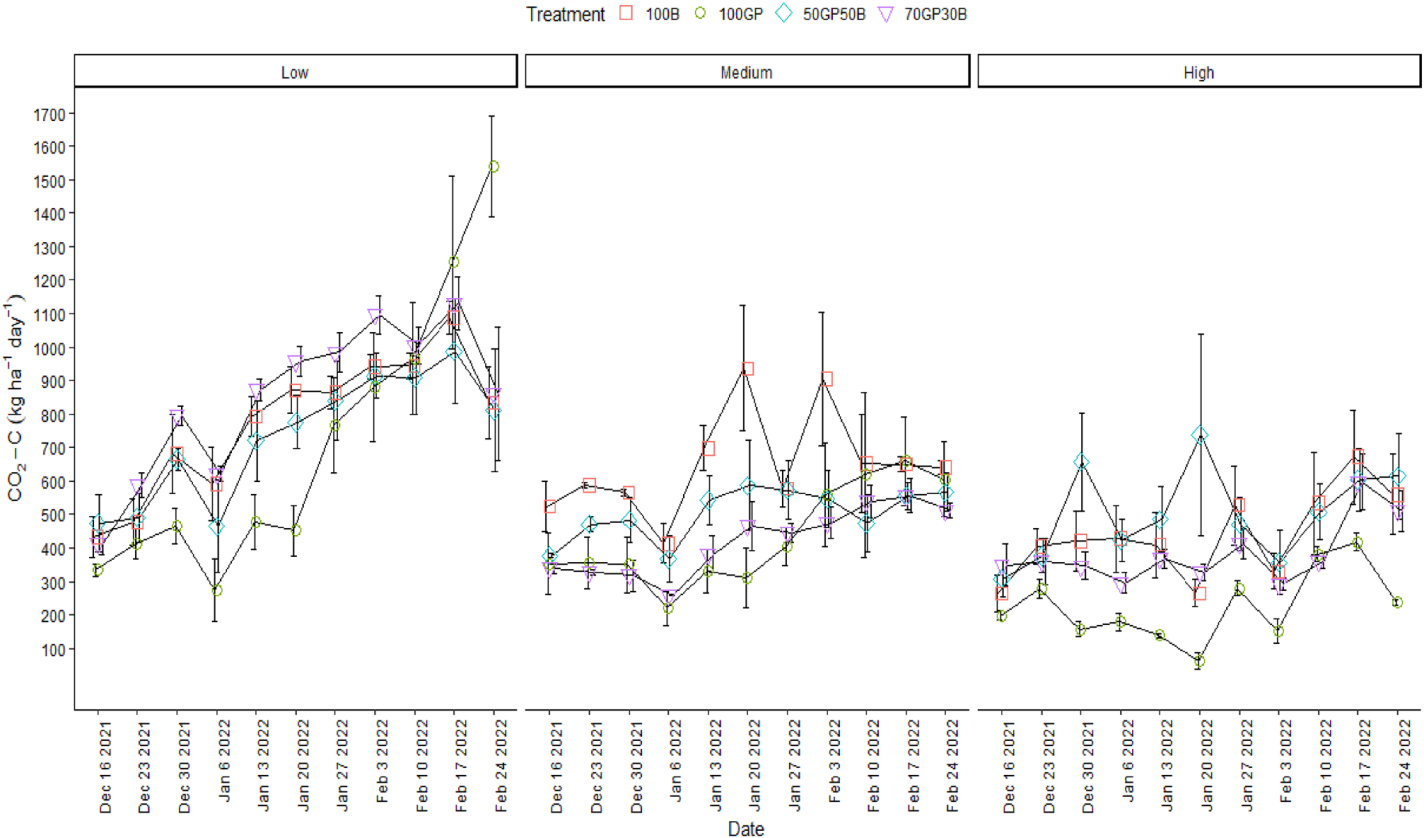
CO_2_-C flux associated with grass pea (GP) and barley (B) monocrops and their mixtures established in 50–50 and 70–30% seeding ratios in low, medium and high soil salinity. Error bars indicate standard error following LSD Fisher protected mean comparisons (p < 0.05).

There was a significant salinity × treatment × observation date interaction (p < 0.0001) to influence soil CO_2_-C flux (Table 1). As shown in Fig. 2, the 70–30% mixture of grass pea and barley mixture maintained the highest CO_2_-C flux during eight out of the eleven weeks of observation. Except for the case initially, this mixture activated soil CO_2_-C release at margins slightly greater than that associated with barley monocrop but significant compared to the counterpart 50–50% mixture and grass pea monocrop. A cross examination of barley monocrop and its mixtures with grass pea over time showed a steady increase in CO_2_-C flux during the three weeks of December followed by an interruption caused by precipitation during the first week of January 2023. During this week, an average of 1.7 mm of rain was recorded (Supplementary Appendix 1). The upward trend of CO_2_-C flux resume in the second week of January, increasing to the highest points in the third week of February and thereafter declined unanimously. Grass pea performed least in inducing soil CO_2_-C flux particularly during 5 weeks of observation but thereafter increased dramatically surpassing barley monocrop and mixtures in February. After the precipitation in February, CO_2_-C emission from grass pea took relatively longer time to recover unlike the other crops.

**Table 1:** Analysis of variance to determine the effects of grass pea and barley monocrops and their mixtures in 50–50 and 70–30% seeding ratios against soil properties and crop yields.

Source of variation	CO_2_-C Flux	VMC	WFPS	EC_e_	Na conc.
Time	23.63***	13.85***	5.58***	131.06***	199.30***
Time × site	22.49***	52.08***	29.35***	394.73***	617.73***
Time × treatment	3.57***	1.55*	0.44^ns^	5.07***	9.20***
Time × site × treatment	2.04***	1.08^ns^	0.47^ns^	4.37***	7.08***

	POXC	DM			
Site	3.01^ns^	27.50***			
Treatment	0.09^ns^	0.60^ ns^			
Site × treatment	0.20^ns^	5.70***			

*VMC* volumetric moisture content, *WFPS* water filled pore space, *EC*_*e*_ electrical conductivity, *Na Conc.* sodium concentration, *POXC* permanganate oxidizable carbon, *DM* forage dry matter yield.

***F significant (p < 0.0001); *F significant (p < 0.05); ^ns^not significant (p > 0.05).

In the site with background medium salinity, barley triggered the release of greater amounts of soil CO_2_-C and was followed in magnitude by the 50–50% mixture of grass pea and barley. Unlike in conditions of low salinity, the 70–30% mixture maintained relatively lower CO_2_-C flux that was to a larger extent comparable to that associated with grass pea monocrop. Also, this site had undulating trends of CO_2_-C flux particularly from soils under mixtures and grass pea, but barley monocrop had spikes in respiration during third week of January and first week of February. It was also worth noting that CO_2_-C flux owing to grass pea monocrop growing in conditions of medium soil salinity changed to less extend for consecutive six weeks but picked up thereafter. This was almost a replicated pattern in low salinity with the exception of the partial increase later in the season.

In the site with highly saline soils, the 50–50% mixture of grass pea and barley had drastically alternating CO_2-_C fluxes which were maximum during the end of December 2021, mid-January and at the end of February 2023. The failure of grass pea to establish in this site confounded the behavior of respiration in that barley monocrop and its mixture with grass pea (50–50%) tended to have moderate temporal variations in CO_2_-C flux but mounted during February. On the other hand, grass pea monocrop maintained substantially lower CO_2_-C emission even though this fluctuated later in the season compared to the rest of the cropping systems. The range of CO_2_-C flux after January 2022 remained at a lower scale in soils with medium and high salinity compared to low salinity.

There were no significant interactions (p = 0.94) witnessed between salinity regime and crop combinations in their influence against soil active carbon (permanganate oxidizable carbon). Likewise, salinity had no significant influence (p = 0.06) against soil active carbon. For that matter, active carbon ranged from 94, 168 and 204 mg kg^−1^ in soils of high, low and medium salinity respectively (Table 2). Even so, there was a pattern indicating that the 70–30% grass pea-barley mixture had consistent and greatest levels of soil permanganate oxidizable carbon detected in low, medium, and high soil salinity (196, 220, and 130 mg kg^−1^).

**Table 2:** Permanganate oxidizable carbon in soils planted with grass pea (GP) and barley (B) monocrops and their mixtures established in 50–50 and 70–30% seeding ratios in sites with a range of low to high soil salinity.

Treatment	Permanganate oxidizable carbon			Mean
	Low salinity	Medium salinity	High salinity	
	mg kg^−1^ soil			
100B	145	208	98	150
100GP	155	213	61	143
50GP50B	177	165	85	142
70GP30B	196	229	130	185
Mean	168	204	94	

### Influence of barley–grass pea mixtures on soil physical properties

**Figure 3:**
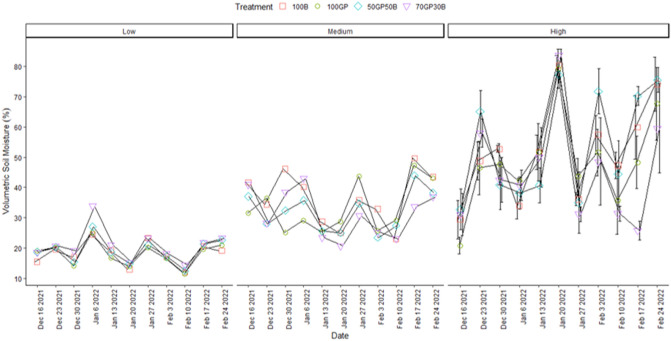
Volumetric moisture content in soils beneath grass pea (GP) and barley (B) monocrops and their mixtures in 50–50 and 70–30% seeding ratios during December 2021 to February 2022. Error bars indicate standard error following LSD Fisher protected mean comparisons (p < 0.05).

**Figure 4:**
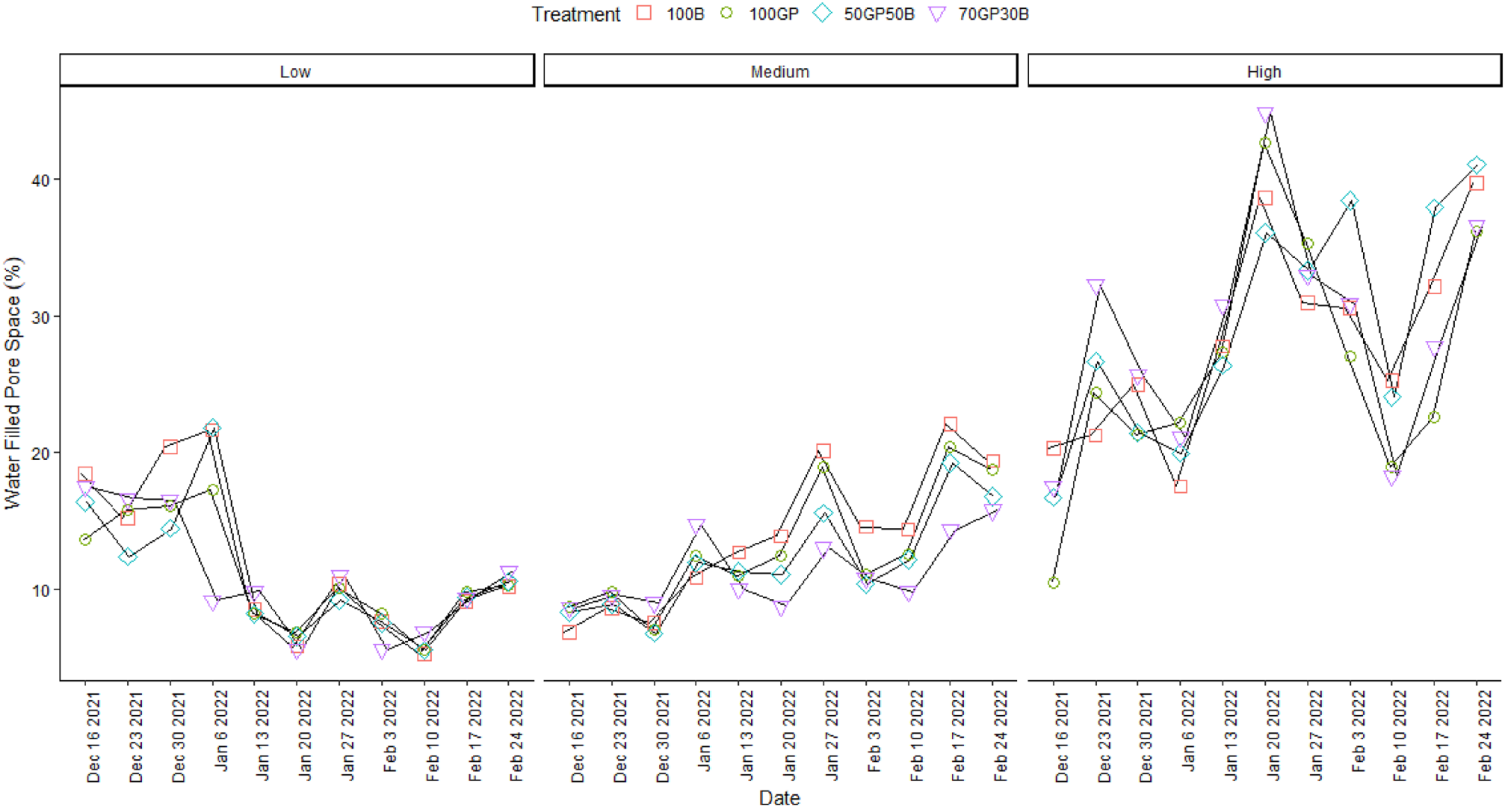
Water filled pore space of soils beneath grass pea (GP) and barley (B) monocrops and their mixtures in 50–50 and 70–30% seeding ratios during December to February 2022. Error bars indicate standard error following LSD Fisher protected mean comparisons (p < 0.05).

There was no significant interaction between date, site and treatment (p = 0.34) to influence volumetric soil moisture (Table 1). It is only in the location with high salinity that moisture varied across treatments over time (p = 0.02). In this case, the 50–50% grass pea, barley mixture tended to retain greater moisture during peak moisture recharge and in some occasions depleted soil moisture to relative lower contents compared to the other treatments (Fig. 3). Moisture fluctuated sharply over the entire season in highly saline soils and remained greater compared to observations in medium salinity while less pronounced effects were evident in soils with low salinity. As expected, volumetric moisture content had strong correlations to water filled pore space in grass pea (R^2^ = 0.9) and barley (R^2^ = 0.7) monocrops and their mixtures (R^2^ = 0.7). Over time, water filled pore space was not significantly influenced (p = 0.9) by treatments across sites. But there were distinctions in soil water filled pore space along the gradient from medium to high soil salinity. Highly saline soils were dominated with water filled pores compared to medium to low salinity (Fig. 4). The difference widened during mid to end season during January to February. During similar period, soils with medium salinity had intermediate water filled pore space which exceeded the amounts in low salinity.

### Influence of barley–grass pea mixtures on soil salinity and sodium concentration

**Figure 5:**
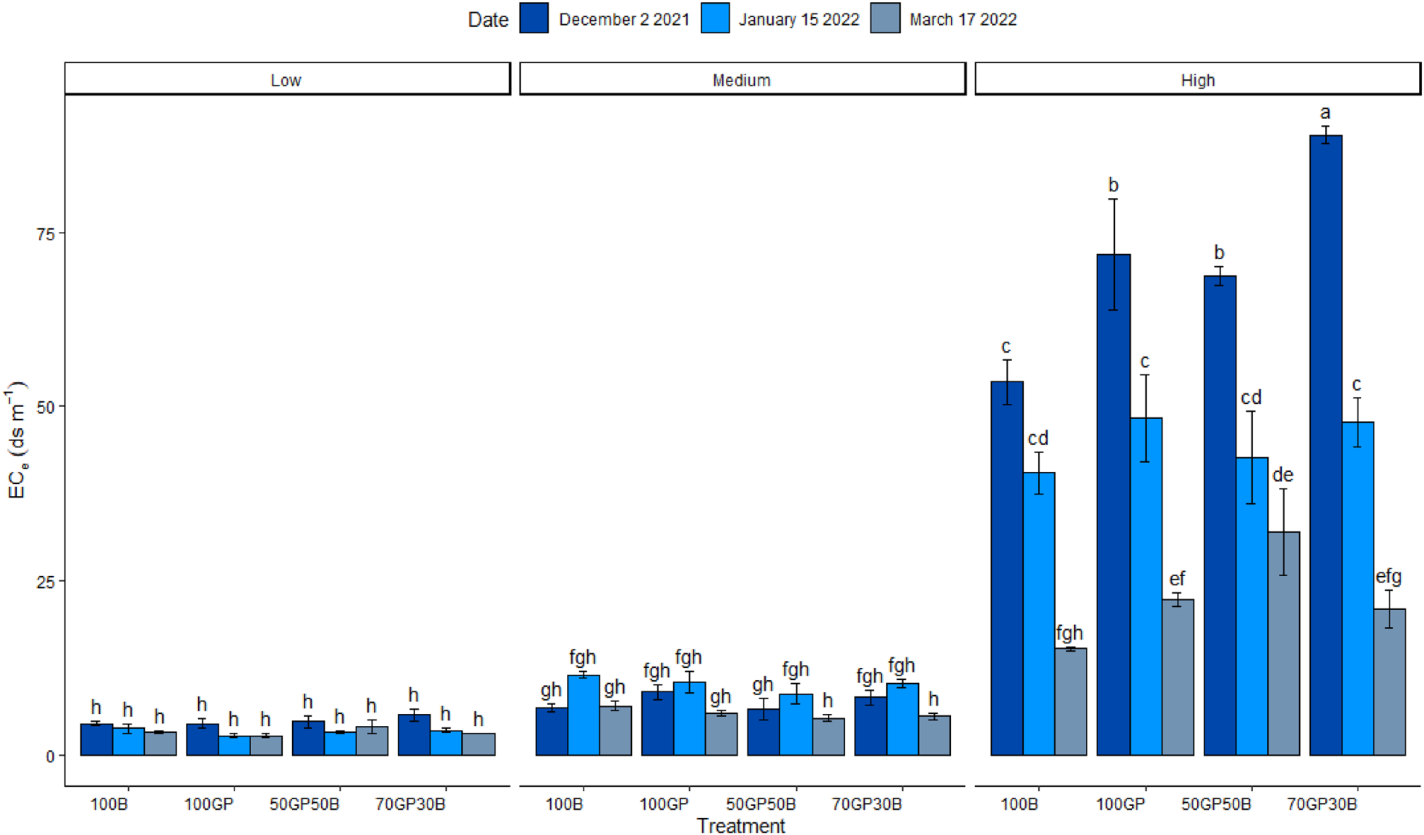
Electrical conductivity of soils beneath grass pea (GP) and barley (B) monocrops and their mixtures in 50–50 and 70–30% seeding ratios established in soils of low, medium and high salinity during December 2021 and January and March 2022. Error bars indicate standard error following LSD Fisher protected mean comparisons (p < 0.05).

Site × treatment interacted over time (p < 0.0001) to influence soil salinity (Table 1). In sites with inherently low and high salinity, soil EC_e_ reduced consistently across time of the season regardless of treatment (Fig. 5). In originally low salinity, the 70–30% grass pea, barley mixture had relatively greater reduction in soil EC (6 to 3 dS m^−1^) compared to the 50–50% mixture (5 to 4 dS m^−1^) but similar range as monocrops (5 to 3 dS m^−1^). In inherently highly saline conditions, soils planted with grass pea monocrop (72 to 22 dS m^−1^) and 70–30% mixture of grass pea and barley (89 to 21 dS m^−1^) had the most pronounced change in EC_e_, unlike the 50–50% barley, grass pea mixture (69 to 32 dS m^−1^) and barley monocrop (54 to 15 dS m^−1^). Conversely, in inherently moderately saline soils, EC_e_ increased across treatments during mid-season (January) but eventually reduced (December). However, the 50–50% barley, grass pea mixture, reduced soil EC to the least level at the end of the season (7 to 5 dS m^−1^). At the end of the season, salinity was found to be slightly lower compared to the beginning of the season in soils beneath the 70–30% grass pea, barley mixture (8 to 6 dS m^−1^) and monocrops (12 to 6 dS m^−1^).

**Figure 6:**
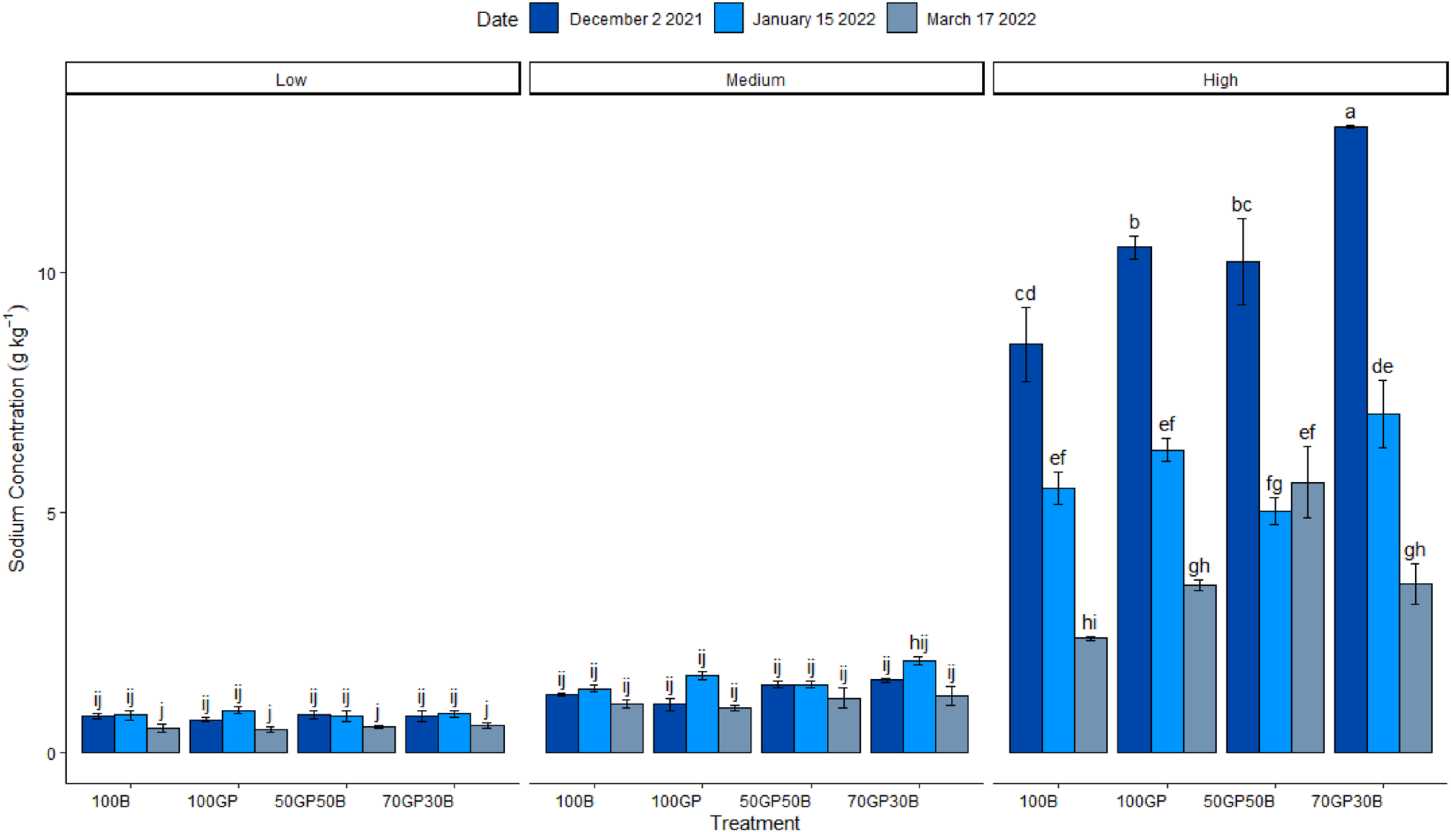
Sodium ion concentration in soils beneath grass pea (GP) and barley (B) monocrops and their mixtures in 50–50 and 70–30% seeding ratios established in soils of low, medium and high salinity during December 2021 and January and March 2022. Error bars indicate standard error following LSD Fisher protected mean comparisons (p < 0.05).

There were significant interactions (p < 0.0001; Table 1) between date, treatment and salinity regimes in their influence against Na^+^ ion concentration in soil. Sodium ion concentrations reduced consistently across the season in soils under all test crops (Fig. 6). However, soils under the 50–50% mixture of barley and grass pea retained relatively more sodium at the end (4.9 g kg) compared to final concentrations in the rest of treatments (3.2 to 3.5 g kg). In soils of medium salinity, sodium ion concentration of soils beneath barley monocrop increased slightly mid-season (1.4 to 1.6 g kg^−1^) but decreased again to 1 g kg^−1^, lower than at the beginning of the season. Grass pea monocrop (1.4 to 1.9 g kg^−1^) and the 70–30% mixture of grass pea and barley (1 to 1.6 g kg^−1^) did not reduce, but had enhanced sodium salt concentration mid-season. While the 50–50% grass pea, barley mixture had only slight change of sodium (1.3 to 1.1 g kg^−1^) across the season. Overall, all treatments had a net negative accumulation of salts ultimately compared to the beginning of the season. Grass pea monocrop growing in low salinity, had similar behavior of enhancing sodium concentration mid-season from 0.7 to 0.9 g kg^−1^ similar to its effect in soils of medium salinity. Nevertheless, across treatments, there were lower sodium concentration at the final evaluation 0.5 g kg^−1^ compared to the beginning of the season (0.8 g kg^−1^).

### Relationship between soil moisture and CO_2_-C flux

**Figure 7:**
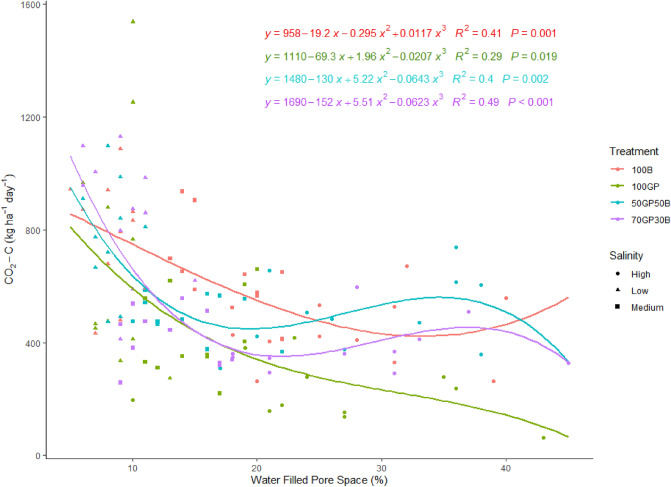
Relationship between water filled pore space and CO_2_-C flux from soils beneath grass pea (GP) and barley (B) monocrops and their mixtures in 50–50 and 70–30% seeding ratios established in soils of low, medium and high salinity.

There were no significant correlations (p > 0.05) between volumetric soil moisture content and CO_2_-C flux in each of barley and grass pea monocrops and their mixtures. However, there were significant (p < 0.05) polynomial regression models describing the relationships between water filled pore space and soil respiration (Fig. 7). Across a gradient from low to moderate salinity, barley-grass pea mixtures had similar patterns of rapidly reducing CO_2_-C flux along a range of increasing water filled pore space upto 15%. Enhanced water filled pore space above 35% coincided with increased CO_2_-C flux. CO_2_-C flux in soils under barley reduced in a linear manner with increasing water filled pore space to reach a minimum at ~ 35% water filled pore space followed by a slight increase in gas flux. At high soil salinity, CO_2_-C flux increased slightly in response to enhanced water filled pore space above 35%. Barley monocrop had less rapid, linear phase of reducing CO_2_-C flux to a minimum at ~ 35% water filled pore space, over this point, increasing water filled pore space slightly enhanced gas flux. In contrast, across moderate to high salinity, grass pea had consistently reduced CO_2_-C flux across the entire range of increasing water filled pore space.

### Forage dry matter yield

**Figure 8:**
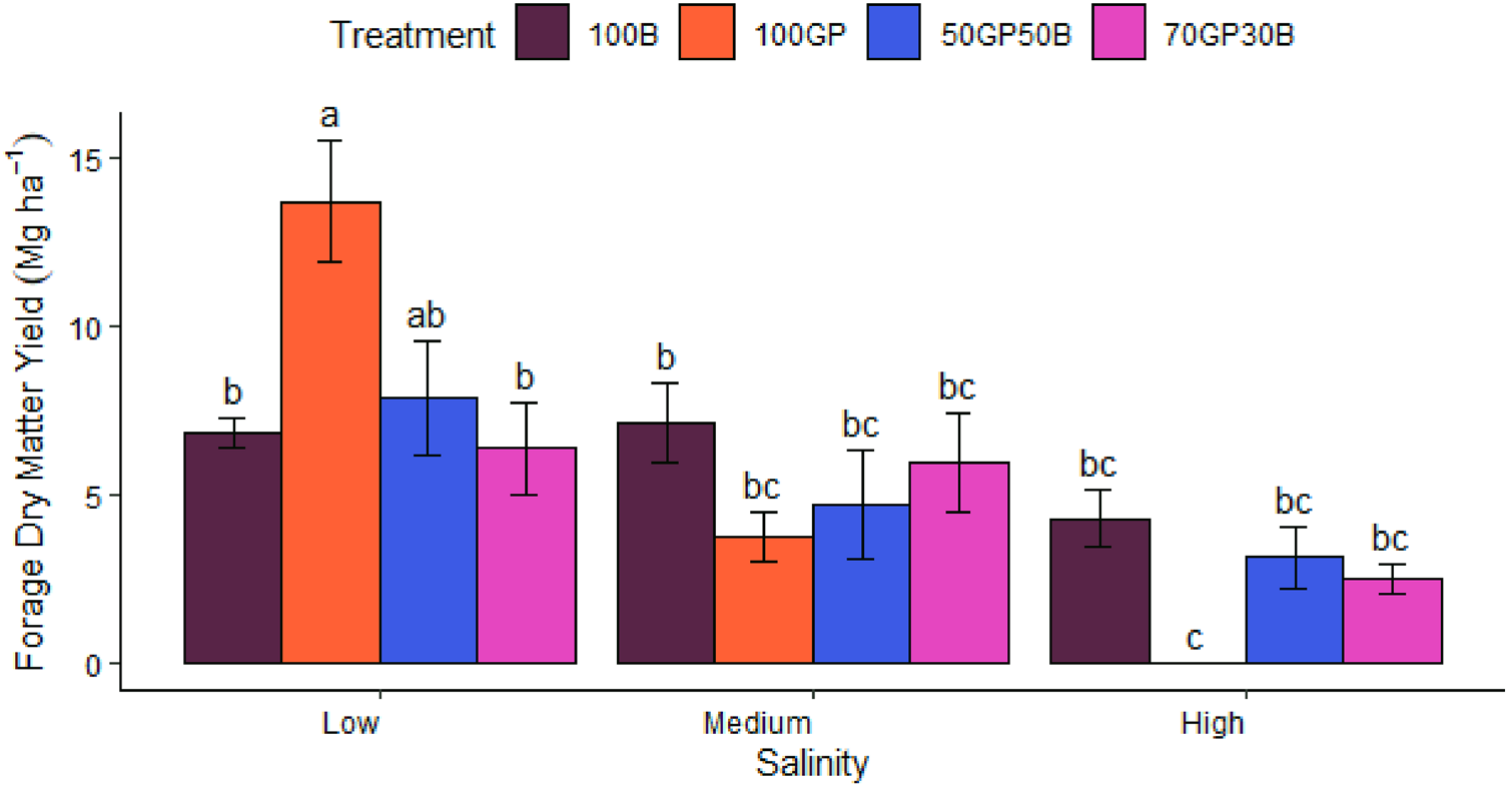
Forage dry matter yield of grass pea (GP) and barley (B) established as monocrops and in 70–30% as well as 50–50% seeding ratios. Yields were obtained in March 2022.

Crop forage dry matter yield varied significantly (p = 0.002) across treatments and soil salinity in different sites. The interactions are shown in Fig. 8. Although this variation was clear in low salinity where against expectation, grass pea had the highest dry matter yield of 13 Mg ha^−1^, followed by the 50–50% mixture of grass pea and barley (8 Mg ha^−1^). The 70–30% grass pea-barley mixture (6 Mg ha^−1^) and barley monocrop (7 Mg ha^−1^) had yields comparable to that of the 50–50% mixture, but significantly lower than that of grass pea monocrop. In medium salinity, barley dry matter yield marginally surpassed that of the 70–30% grass pea-barley mixture and in the same order that of the counterpart 50–50% mixture and grass pea monocrop. In conditions of high salinity, again barley monocrop generated the greatest dry matter followed by the 50–50% mixture and the 70–30% grass pea-barley mixture. However, in this site, grass pea did not survive beyond the seedling stage.

### Influence of crop shoot dry matter and soil active carbon against CO_2_-C flux

**Figure 9:**
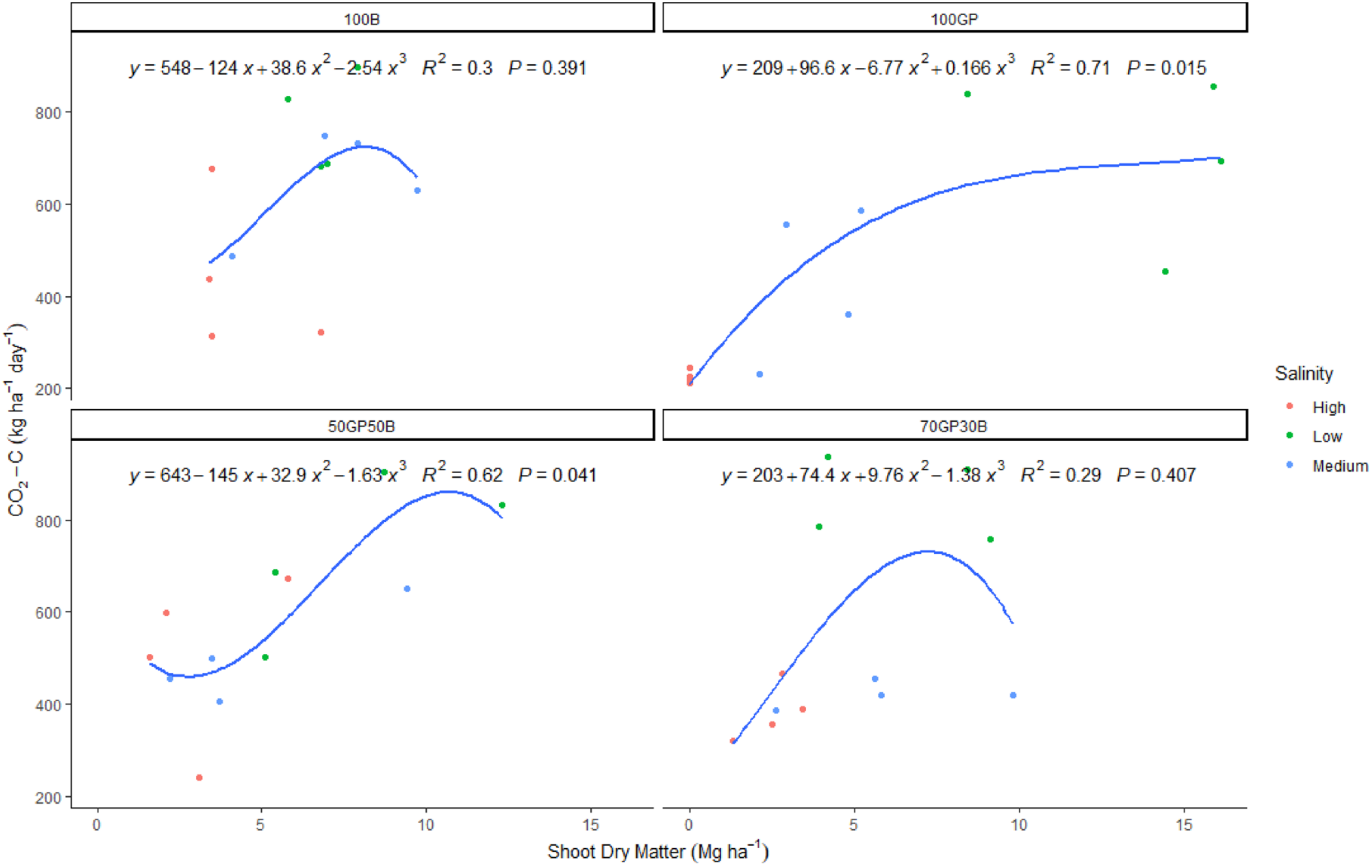
Polynomial regression of the influence of shoot dry matter yield on CO_2_-C flux under grass pea and barley monocrops and binary mixtures established in sites with varying soil salinity and averaged during December 2021 to February 2022.

Regression models indicate significant cubic relationships (y = 209 + 96.6x − 6.8x^2^ + 0.17x^3^, R^2^ = 0.7, p = 0.02) between shoot dry matter of grass pea monocrop and soil CO_2_ flux (Fig. 9). A slightly different model was fitted for the relationship between shoot dry matter of the 50–50% mixture of barley and grass pea (y = 643 − 145x + 32.9x^2^ − 1.63x^3^, R^2^ = 0.6, p = 0.04). There were no significant (p > 0.05) linear or quadratic relationships between shoot dry matter of barley monocrop and the 70–30% mixture of grass pea and barley crops in their individual influence against soil CO_2_-C flux. There were also no significant correlations between soil active carbon and CO_2_-C flux associated with barley and grass pea mixtures or barley monocrop. Nevertheless, grass pea monocrop had exceptional significant positive linear relationship (y = 149 + 2x, R^2^ = 0.39, p = 0.031) between soil active carbon and CO_2_-C flux.

## Discussion

Results of this experiment confirm that mixtures can enhance soil microbial activity. This synergy was stronger in soils with inherently low salinity, but diminished along the gradient of increasing salinity. We established that unlike non saline conditions, carbon mineralization in saline-desertic conditions is controlled by a more complex interaction of crop salinity tolerance, crop water relationship and the confounding effects of shallow soil profiles. To begin with the crop factors, it was clear that microbial respiration was sensitive to the thriving of both barley and grass pea. In fact, although grass pea growing alone took longer to induce rapid soil CO_2_-C flux, it eventually surpassed the effects of barley monocrop and mixtures under less severe salinity. It is generally known that a mixture of a cereal and a legume crop can attract a diversity of microbes and at the same time provide the energy for their metabolism in root exudates. More precisely, mixed species can have additive effects by providing different sources of carbon to microbes along with dissolved nitrogen^19^. It was also worth noting that the 70–30% mixture of grass pea and barley was observed to enhance soil active carbon across environments upto extremely saline conditions. In reality, we observed that grass pea growth was invigorated when dominating in mixtures. This legume also intensified CO_2_-C flux to surpass that of mixed cropping systems and barley monocrop. The gains in microbial activities under influence of mixtures can be explained by the correlation of leaf photosynthetic carbon assimilation and root vigor and active carbon content^20^. When CO_2_-C flux of grass pea monocrop was fitted against shoot dry matter, it was estimated that approximately 5 Mg ha^−1^ of shoot dry matter was the critical point below which microbial respiration reduced sharply as a result of increasing salinity. Positive relationships were also observed in the 50–50% barley, grass pea mixture shoot dry matter and soil CO_2-_C flux albeit, above 10 Mg ha^−1^ of shoot dry matter, respiration diminished irreversibly. Unlike barley whose photosynthesis is sustained by leaves, grass pea has the ability to retain photosynthetic stems even after leaves wither due to salt toxicity^21^. On the other hand, barley photosynthesis has long been associated with promoting soil microbial respiration which reaches the peak at flowering stage^22^.

Undoubtably, grass pea resilience against salinity can enrich soils and strengthen the benefits of mixtures. Surprisingly in low salinity grass pea yield exceeded that of barley by almost half. Conversely, grass pea hardly survived beyond the seedling stage following persistent irrigation with extremely saline water (17 dS m^−1^) and a range of soil salinity between 20 and 71 dS m^−1^ across the season. In moderately saline soils, even after adequate growth upto flowering stage grass pea succumbed to acute levels of salinity following excessive soil wetting. Grass pea is known to tolerate moderate salinity. According to Ref.^23^, salinity equivalent to 15 dS m^−1^ can reduce emergence of grass pea by 35% while further mortality of seedlings occurs at as low as 10 dS m^−1^. We observed that, abrupt and excessive wetting of soils after effective precipitation recorded in January deposited sodium in the root zone on subsequent drying. Under such circumstance, plants absorb greater amounts of sodium which builds up in the cell vacuole while potassium uptake is suppressed^24^. Potassium plays a key role in plant metabolism including maintaining osmotic potential, stomatal conductance and photosynthesis. Therefore, with the imbalanced supply of potassium, grass pea shoot growth is arrested. It was also manifested that salt damage to grass pea encouraged the competitive advantage of barley in close proximity in mixtures. During the rest of the season, grass pea did not recover to these stresses.

The aforementioned challenges of grass pea establishment in mixtures reduced any possible chance that barley can alleviate salinity and allow grass pea to thrive. Generally, salinity and sodium concentrations in soils tended to reduce at the end compared to the beginning of the season regardless of treatments. We also take cognizance of the fact that in highly saline soils persistently irrigated with extremely saline irrigation water, there were sharp gradients of salinity and sodium losses across the season. This is likely because of the shallow soil profile embedded with rocky fragment which kept the soil saturated with saline water in the previous season. Thereafter, there was approximately three months of fallow and dry weather without further irrigation before the test crops were established. This exposes the stored water near the soil surface to evaporation leaving behind unprecedent amounts of salts. This is a typical occurrence in areas with saline water and shallow ground water salinity without allowance for leaching^25^. It was therefore possible to accelerate the exclusion of the accumulated salts from the rooting zone on resumption of drip irrigation. Notwithstanding that salinity reduced across treatment, barley maintained relatively lower salts in the soils throughout the season. This still sheds some light on the potential ameliorative role of barley against severe salinity. Barley is endowed with salt tolerant mechanisms even though this crop suffers some penalties on shoot dry matter accumulation. The prominent trait of barley is its ability to absorb and sequester sodium in root vacuoles^26^. In the site with moderately saline soils, salinity surged during mid-season across different cropping systems. This was much shallower soil profile compared to the highly saline soils with a bedding of rock fragments at approximately 15 cm below the soil surface and in some areas, there were patches without this layer. This is why the time series of volumetric moisture content in this site was inconsistent. The series of irrigation events and relatively low evapotranspiration in the cold months of January and February maintained water near the soil surface. But in few occasions when there was intense sunshine, this water escaped and precipitated salts in the soil surface. In moderate to low salinity, it was observed that barley monocrop and its mixture with grass pea in 50–50% seeding ratios had less pronounced change of sodium concentration in the soils during mid-season. This again suggested that barley might be offloading some considerable amounts of salts accumulating in the soil during active vegetative growth. Salinity and sodium concentration remained lowest in the site with initially low salinity. This site has a deeper layer dominated by loose sandy soil upto ~ 32.5 cm. Despite the fact that this site had irrigation water with salinity at 8 dS m^−1^, much of the salt can be drained down through the loosely soil profile.

It was established from correlations that soil CO_2_-C flux depended on the soil water–air balance and not purely soil moisture. But it was also revealed that soil moisture influenced crop establishment and by proxy soil respiration. We found results contrary to the general sigmoid pattern where microbial respiration increases with water filled pore space but decelerates as anaerobic conditions set in Ref.^27^. Instead, CO_2_-C flux reduced steadily with increasing water filled pore space and salinity to approximately 20% and recovered slightly at 35% water filled pore space under influence of barley-grass pea mixtures. Same pattern of decreasing soil respiration applied to barley to reach the minimum level at ~ 35% water filled pore space. At greater water filled pore space and salinity, the model indicated that respiration recovered again. It is possible that under conditions of low to moderate salinity, soil microbial respiration endures osmotic stress associated with increasing content of saline water in the soil pore spaces. But at extremely high salinity, as we noted earlier, barley might be extracting salts and therefore providing niches with diffused osmotic stress against microbes. The diversity of species mechanisms can create diverse soil microhabitats that segregates soil microbial communities allowing some to proliferate and mineralize organic matter^28^. In the fitted model, we identified the irreversible consequences of increasing water filled pore space and salinity to microbial activities in soils under grass pea. This conformed to the pattern of reducing grass pea dry matter productivity as salinity increased. This emphasized the loss of grass pea ecosystem services to sustain soil microbial activities as salinity intensified. Nevertheless, soils beneath grass pea maintained some minimum respiration despite the high salinity. We therefore form the view that, CO_2_-C flux in these soils might be under the influence of factors secondary to crop biological activities. The highly saline soils had 14% clay compared to 12% at medium and low salinity. Discursive to the slightly revived CO_2_-C flux under high salinity, an abundance of sodium salts tends to disperse wetted clay minerals and therefore expose encrusted organic matter to dissolution in soil water^9^. The dissolved organic matter serves as substrate providing energy for soil microbial activities^29^.

The results of this study revealed no obvious signs of soil microbial activities alleviating soil compaction hence indirectly reducing salinity. Although at the onset, it was envisaged that robust microbial activities stimulated by dense root growth of barley–grass pea mixtures might increase soil aggregation and reverse the effects of salinity on soil compaction. There were only slight indicators that barley grass pea mixtures could encourage accumulation of soil active carbon, but this was not enough to influence soil quality. The tendency to exploit above ground crop residues in arid areas of Morocco denies the soil microbes organic substrates enough to facilitate processes of soil aggregation. Instead, Ref.^30^ have recorded greater impact of crop residue addition to aggregation of saline soils than that associated with innate root-microbe interactions. They identify stronger influence of humic products of decomposing straw in enhancing porosity, hydraulic conductivity and exclusion of sodium from soils rather than indirect mechanism of microbial polysaccharides. In addition, the organic matter of the soils in this study remained below the 2% threshold above which organic compounds have pronounced effects of soil aggregation^31^.

## Conclusions

Soil salinity and sodicity can adversely affect crop establishment and restorative microbial activities. This study was set up with the goal to identify mechanisms of irrigated barley and grass pea mixtures to alleviate salinity and support microbial regeneration of saline soils. It was apparent that barley can alleviate sodium accumulation and alleviate salinity upto extreme levels. It was demonstrated that dominance of grass pea at low salinity can boost productivity in 50–50% mixtures with barley, but these benefits were devastated as salinity increased. The 70–30% mixture of grass pea and barley had the potential to accelerate carbon mineralization simultaneous with stabilization. We found that crop establishment and microbial activity in the desert environment was confounded by impeding layers of rocks near the soil surface and rapid salinization of surface soils by evaporating water. It is deduced that this challenge can be everted by moderating irrigation relative to increasing soil salinity and shallow soil profiles.

## Materials and methods

### Experimental site description

Field experiments were conducted in three sites in Foum El Oued located in the Southern region of Morocco. The sites included low, medium and high salinity platforms, located at coordinates 27° 11′ 01.2′′ 13° 19′ 29.4′′ W; 27° 11′ 30.2′′ N 13° 19′ 43.2′′ W and 27° 11′ 10.6′′ N 13° 20′ 28.9′′ W, respectively. The corresponding salinity of saturated soil pest extract (EC_e_) were 8.8, 25.5 and 54.4 dS m^−1^. The EC_e_ values were derived from EC_1:5_ values of 1.1, 3.3 and 7.1 dS m^−1^ based on a regression Eq. (1). The salinity of irrigation water respective to these sites was 8, 4 and 17 dS m^−1^. As indicated in Table 3, soils across sites had sandy loam texture with slightly alkaline pH (7.7 to 7.9) and low soil organic C (1.2 to 1.7%).

**Table 3:** Initial soil physical and chemical properties at different sites.

Parameter	Site		
	Low salinity	Medium salinity	High salinity
Textural class	Sandy loam	Sandy loam	Sandy loam
Sand:silt:clay ratio (%)	68:20:12	60:28:12	60:26:14
Organic carbon (%)	1.6	1.7	1.2
Total nitrogen (%)	0.1	0.1	0.1
EC_e_ (dS m^−1^)	8.8	25.5	54.4
pH_1:5_ (1:5)	7.9	7.8	7.7

### Experimental design and establishment

Experiments were set up in the randomized complete block design with four treatments nested within each site. Treatments included four forage cropping systems specifically grass pea and barley monocrops and their mixtures in 50–50 and 70–30% seeding proportions. Treatments were replicated four times. Seeds of barley were obtained from the National Institute for Agricultural Research (INRA)-Benimellal and grass pea from the International Center for Agricultural Research in the Dry Areas (ICARDA), Rabat, Morocco in 2021. The UM6P has general agreements with INRA and ICARDA in Morocco that include transfer and sharing of material including crop seeds, reagents and equipment for research purposes. Therefore, all the seeds obtained for the experimental research conducted in this study and all the methods used comply with relevant institutional, national, and international guidelines and legislation.

Seeding rates and ratios were estimated based on pure live seed procedure^32^. This is an estimate that adjusts the requisite quantity of seeds to a value higher than recommended after accounting for the shortfall from 100% seed germination and purity. The recommended seeding rates of barley and grass pea were 150 and 50 kg ha^−1^, respectively. Proportionate quantity of seeds in mixtures were computed by multiplying seeding ratios with pure live seed values. Seeds were mixed thoroughly for bicultures before planting. Each experimental plot measured 13.5 m^2^. Three months before establishing experiments, all fields were fallow. But before that, the site with low and medium salinity had histories of three fallow seasons, while that in high salinity had a durum wheat crop. Soils were tilled twice with a disc plough to a medium fine tilth. Thereafter, drip lines were installed in each site to laterals receiving water from basins supplied with borehole water. Seeding was implemented by hand drilling on November 22, 2021. Seeding rows were 0.5 m apart and each aligned with a preinstalled drip irrigation line. All sites were planted within a span of four days. Irrigation started immediately after planting in respective sites. Crops were irrigated according to a water budget guided by crop water evapotranspiration and approximately 50% maximum allowable soil water depletion and effective precipitation (Supplementary Appendix 1, Table 1). Hence, early in the first three weeks of crop establishment, irrigation rates varied from 10.5 mm in each event, twice per week. Subsequently rate was increased to 14.5 mm per event upto the 13th week. Thereafter amounts of water per irrigation was reduced to 7 mm per event along with crop physiological maturity. Plots were maintained weed free by a preemergence spray of glyphosate and two hand tillage operations at five and nine weeks after crop establishment.

### Soil chemical analysis

Soils were sampled to determine soil chemical properties. Initially, before planting, soils were sampled at five random spots in each plot and blended for determination of pH, EC, organic carbon, sodium and total nitrogen. An additional set of samples were collected at 15 cm depth in December 2, 2021, January 15 and March 17, 2022 to determine EC, and concentration of sodium and potassium. Soils were taken to the African Sustainable Agriculture Research Institute Soil Laboratories for analysis. Soils were mixed with water in 1:5 ratio for determination of pH and EC_1:5_ values. EC_1:5_ values were converted to equivalent saturated paste extract (EC_e_) according to Eq. (1)^33^.

1 $$\mathrm{ECe}=\left(7.46\times \mathrm{ EC}1:5\right)+0.43$$

Soil organic matter was analyzed using the weight loss-on-ignition method^34^. Soil K and Na concentrations were determined by ammonium acetate extraction procedure and flame photometry^35^.

### Soil microbial respiration and physical properties

Soil microbial respiration was measured weekly using a portable CO_2_ gas analyzer (EGM-5, PP Systems -USA) connected to a soil respiratory chamber (SRC) (150 mm height and 100 mm diameter) and Hydra Probe II-Stevens Water Monitoring System. During each event of gas analysis, the soil respiratory chamber was lodged onto a 10 cm height × 10 cm diameter PVC rings. After seedling emergence, PVC rings were inserted into the soil at a position mid between crop rows, and to a depth that leaves a 1 cm collar for insertion of the SRC. Gas collection events started two days after installation of PVC chambers each scheduled at around 9.30 am and continued to 11 a.m. for one site. In the next day gas was collected in the other sites during similar time. Air in the SRC was flushed for 20 min before insertion in the PVC collar. A 9 s delay was allowed to stabilize conditions in the enclosed chamber after which, CO_2_ flux was measured at the beginning and 4 min later. Along with gas measurements, soil volumetric moisture content (*θ*_*V*_) and soil and air temperatures were recorded at the end of each gas sampling event using the Hydra Probe II-Stevens Water Monitoring System inserted close to each gas sampling station.

Respiration rate was calculated according to Eq. (2)^36^.

2 $${F}_{{\text{CO}}2}\left({\text{kg}}\, {\text{ha}}^{-1} {\text{day}}^{-1}\right)=\left[\frac{dC}{dT} \times \frac{P}{1013}\times \frac{273}{273+Tair}\times \frac{44.009 }{22.414 } \times \frac{\mathrm{V }}{\mathrm{A }}\right]\times 24 \times 10$$

where $\frac{dC}{dT}$ is the linear regression of CO_2_ flux against time during each gas measurement; $\frac{P}{1013}$ is correction for barometric pressure where P represents the detected air pressure during each gas analysis; $\frac{273}{273+Tair}$ is the correction for air temperature (*Tair*); $\frac{44.009}{22.414}$ is the molar volume and Ideal Gas constant at standard temperature and pressure; V is the chamber volume (0.001171 m^3^), and A its surface area (0.0078 m^2^); 24 and 10 were applied to convert gas flux to rates ha^−1^ day^−1^.

Soil bulk density and penetration resistance were measured at the end of each season. For penetration resistance, a penetrometer (Model: FieldScout SC900, Spectrum Technologies Inc., USA) was used. Gravimetric moisture content and water filled pore space were determined to 15 cm soil depth in each plot. Bulk density was determined from oven dry weight of soil cores collected at 0 to 15 cm, each plot divided by volume^37^. Water filled pore space was determined as the ratio between soil volumetric moisture content and percent total soil porosity^38^. Soil porosity was derived as percentage fraction of bulk density over total particle density (2.6 g cm^−3^).

### Statistical analysis

The analysis of variance (ANOVA) was conducted using the general linear model procedure of stat package, in R software version 3.6.3.^39^. The statistical model adopted was according to a RCBD with date of sampling, salinity, treatment, block, and date of sampling × salinity × treatment as fixed terms. In addition, date of sampling was analyzed as a repeated measure. Means were compared in the *Post-hoc* test using Fisher’s protected LSD (α = 0.05). The R software version 3.6.3 was also used to test the significance of regression coefficients describing the relationships between individual predictor variables including soil moisture and salinity, water filled pore space, shoot dry matter, versus CO_2_-C flux in different crops under evaluation. Means and regression models were visualized using the ggplot2 package in R software version 3.6.3^39,40^.

### Supplementary Information

Supplementary Information.

## Data Availability

The data that support the findings of this study are available from the corresponding author upon reasonable request.
