# Supplementary material for: Irrigated barley–grass pea crop mixtures can revive soil microbial activities and alleviate salinity in desertic conditions of southern Morocco
Source: Sci Rep. 2023 Aug 14;13:13174. doi: 10.1038/s41598-023-40337-9 (PMC10425461; doi:10.1038/s41598-023-40337-9)
Supplement: Supplementary file 1 — Supplementary Information. [file 41598_2023_40337_MOESM1_ESM.docx]

**Appendix 1**

**Table.1**

|  | **Water Amount** | | | | | | | | | | | | | | | | | | | | | |
| --- | --- | --- | --- | --- | --- | --- | --- | --- | --- | --- | --- | --- | --- | --- | --- | --- | --- | --- | --- | --- | --- | --- |
| **Year** | **2021** | | | | | |  | **2022** | | | | | | | | | | | | | |  |
| **Month** | **Nov** |  | **Dec** | | | |  | **Jan** | | | |  | **Feb** | | | |  | **Mar** | | | |  |
| **Week** | **4** |  | **1** | **2** | **3** | **4** |  | **1** | **2** | **3** | **4** |  | **1** | **2** | **3** | **4** |  | **1** | **2** | **3** | **4** | **Sum** |
|  | **mm** | | | | | | | | | | | | | | | | | | | | | |
| Irrigation | 21 |  | 26 | 26 | 29 | 29 |  | 29 | 29 | 14 | 27 |  | 29 | 29 | 29 | 29 |  | 27 | 14 | 14 | 14 | 415 |
| Precipitation | 0.4 |  | 0.1 | 0.1 | 0.1 | 0.0 |  | 0.1 | 1.7 | 7.4 | 0.7 |  | 0.1 | 0.0 | 0.4 | 1.6 |  | 0.2 | 6.0 | 2.3 | 1.0 | 22.2 |
